# Relationship between *XPD*, *RAD51*, and *APEX1* DNA repair genotypes and prostate cancer risk in the male population of Rio de Janeiro, Brazil

**DOI:** 10.1590/1678-4685-GMB-2017-0039

**Published:** 2017-11-06

**Authors:** Ana Sheila Cypriano, Gilda Alves, Antonio Augusto Ornellas, José Scheinkman, Renata Almeida, Luciano Scherrer, Claudia Lage

**Affiliations:** 1 Instituto de Biofísica Carlos Chagas Filho, Universidade Federal de Rio de Janeiro (UFRJ), Rio de Janeiro, RJ, Brazil; 2 Instituto Nacional de Câncer, Hospital do Câncer I, Rio de Janeiro, RJ, Brazil; 3 Hospital Mário Kroeff, Rio de Janeiro, RJ, Brazil; 4 Hospital Universitário Antônio Pedro, Niteroi, RJ, Brazil; 5 Fundação Oswaldo Cruz, Rio de Janeiro, RJ, Brazil; 6 Faculdades Kennedy, Belo Horizonte, MG, Brazil; 7 Laboratório de Marcadores Circulantes, Departamento de Patologia, Faculdade de Ciências Médicas, Universidade do Estado do Rio de Janeiro, Rio de Janeiro, RJ, Brazil

**Keywords:** Prostate cancer, single nucleotide polymorphism, XPD, DNA repair, gene-environment interaction

## Abstract

Susceptibility to cancer ensues in individuals carrying malfunctioning DNA repair mechanisms. The impact of Single Nucleotide Polymorphisms (SNPs) in key DNA repair mechanisms on risk for prostate cancer was investigated in this case-control study. Samples consisted of 110 patients with confirmed prostate cancer and 200 unaffected men, from Rio de Janeiro, Brazil. *XPD/*Lys751Gln (rs13181), *APEX1*/Asp148Glu (rs1130409), and *RAD51*/G135C (rs1801320) SNPs were analyzed by PCR-RFLP. Allelic and genotypic frequencies were calculated and compared by Chi-Square test. The association between SNPs and clinical/epidemiological data was considered significant by Odds Ratio analysis, with IC95% and a p-value≤0.05. Only the *XPD*/Lys751Gln SNP significantly increased susceptibility to disease in southeastern Brazilian men, with *p*≤0.001 [OR=2.36 (1.46-3.84)], with no association with *APEX1* or *RAD51* SNPs. Combined *XPD*+*RAD51* SNPs were highly associated with the disease, *p*≤0.005 [OR=3.40 (1.32-9.20)]. A Chi-Square significant association between *XPD*/Lys751Gln and Gleason score was also observed (OR=9.31; IC95%=1.19–428.0; *p*=0.022). Epidemiological inquiries revealed that exposure to pesticides significantly impacted the risk for prostate cancer in this population. DNA repair dysfunctions seem to prevail among workers exposed to chemical byproducts to cancer in this specific tissue. Non-invasive genotyping SNPs may help assessment of prostate cancer risk in environmentally exposed populations.

## Introduction

An increasing incidence of prostate cancer (PCa) has been observed in Brazil, associated with longer lifetimes and due to improved diagnosis methods in countrywide information databases. The Brazilian National Cancer Institute (INCA) estimates approximately 61,200 new cases in 2016, with a risk of 61.82 cases per 100,000 men (INCA, 2016). Factors such as hypertension, smoking, alcohol consumption, sedentarism/obesity, hypercholesterolemia, and genetic history were identified as top risk factors for PCa (Bostwick *et al.*, 2004).

Oxidative stress seems to influence the prostatic carcinogenic process because of its expressive association with aging and accumulating damage (Platz and Giovannucci*,* 2006). Likewise, environmentally-borne carcinogenic agents can also damage DNA, and different DNA repair mechanisms have been implicated in alleviating such harmful damages (Pramanik *et al.*, 2011). Single Nucleotide Polymorphisms (SNPs) result from single-nucleotide changes in these genes, influencing expression or function of the affected genes. These changes could be critical concerning DNA repair functions.

The *XPD* (*Xeroderma pigmentosum complementation group D*) gene encodes one of the proteins involved in the Nucleotide Excision Repair (NER) pathway (O’Donovan and Wood, 1993). One of *XPD’s* important SNPs is *XPD*/Lys751Gln (rs13181), in which an A → C base substitution at exon 23 causes a Lys → Gln substitution in codon 751, compromising part of the C-terminal domain of the XPD protein and its full DNA repair capacity (White, 2009).

The APE1 endonuclease removes abasic sites from DNA, and is the top enzyme initiating recognition of damaged sites in human Base Excision Repair (BER), along with its 3′ → 5′ exonuclease, DNA 3′-repair diesterase and DNA 3′-phosphatase activities. APE1 plays a major 3′-phosphodiesterase role in initiating the repair of oxidative-generated single-strand breaks (Ramana *et al.*, 1998). A missense G → T substitution SNP in the *APEX1* gene in exon 5 (rs1130409) changes a critical Asp → Glu residue at codon 148. An up to date report connects this SNP to the development of PCa in Asian descendents (Chen *et al.*, 2016).

The human *RAD51* gene codes for a bacterial RecA recombinase homolog, central to the Homologous Recombination Repair (HRR) mechanism (Galkin *et al.*, 2006). The RAD51 protein is critical for maintaining genomic integrity by repairing DNA double-strand breaks. The *RAD51* polymorphism (rs1801320) implies in a G → C substitution mapping upstream the 5′ untranslated promoter region at -135 bp from the transcription start. Although the functional consequences of this SNP remain to be clarified, a single nucleotide change in this CpG promoter island may up-regulate gene expression, thus affecting *RAD51* mRNA levels (Antoniou *et al.*, 2007). This SNP has been linked to breast and ovarian cancer susceptibility due to the supposed interaction of RAD51 with BRCA1 and BRCA2 proteins, with an especially higher risk for carriers of *BRCA2* mutations (Levy-Lahad *et al.*, 2001).

The investigation on this assembly of SNPs was designed because they significantly impact main DNA repair mechanisms (NER, BER and HRR, respectively) and genetic instability and, under this perspective, had never been addressed in a Brazilian population before. Considering the importance of DNA repair failures in cancer development, the aim of this work was to investigate the contribution of SNPs in key genes *XPD/*Lys751Gln, *APEX1*/Asp148Glu and *RAD51*/G-135C in the susceptibility to PCa in a case-control study in Rio de Janeiro, Brazil.

## Subjects and Methods

### Study population

For this study, 110 patients (62.0 ± 6.5 mean age at diagnostic) who had undergone radical prostatectomy and subsequent chemo- and/or radiotherapy were recruited between 2006-2008 from the National Cancer Institute (INCA) and Mario Kroeff Hospital (Rio de Janeiro, Brazil). The control group consisted of 200 men (61.6 ± 10.3 mean age) undergoing routine tests, without suspicion of PCa, at the Antonio Pedro University Hospital (APUH, Niterói, RJ, Brazil), sampled between 2009-2010. The study was approved by the Ethics Committees of INCA (#91/05) and APUH (#48/09). All participants signed a written informed consent. The recruited patients carried T1, T2, or T3 prostate tumors, according to the TNM scoring system (tumor extension and metastasis).

Individuals included in the study met the clinical criteria of having a palpable mass by digital rectal examination (DRE) and/or elevated PSA serum levels (C > 2.5 ng/mL) indicative of the occurrence of a tumor, followed by histopathological confirmation of PCa. All patients were treated with radical prostatectomy. Gleason scoring was obtained by a pathologist’s biopsy evaluation and PSA levels by clinical blood tests.

Patients and controls were interviewed following a structured individual questionnaire covering educational level, familial cancer and medical history, place and date of birth, place of birth of father and mother, weight, height, and place of residence. We also profiled the occurrence of prostate and other cancers in first-degree relatives, smoking status, and alcohol consumption. Patients and controls were asked to self-declare skin color, which is a normal question in such inquiries in Brazil. Controls and patients were excluded if they showed genetic syndromes and past cancer history.

### Genotyping

Genomic DNA was isolated from 4-5 mL peripheral blood by proteinase K digestion and phenol-chloroform extraction (Sambrook, 1989). The selected SNPs were genotyped by polymerase chain reaction-restriction fragment length polymorphism (PCR-RFLP). The *XPD/*Lys751Gln (rs13181), *RAD51/*G-135C (rs1801320), and *APEX1* Asp148Glu (rs1130409) SNPs were determined following conditions described by Baccarelli *et al.* (2004), Wang *et al.* (2001), and Hu *et al.* (2001), respectively.

*XPD* and *APEX1* amplicons were digested by 10 U of the specific restriction endonucleases (37 °C, 2 h). The *XPD*/Lys751Gln genotypes A/A, A/C, or C/C were profiled as *Pst*I digestion products of 100/224, 66/100/158/224, or 66/100/158 bp bands, respectively. The *APEX1*/Asp148Glu genotypes T/T, T/G, or G/G were profiled as *Bfa*I digestion products of 164, 164/144/20, or 144/20 bp bands, respectively. For *RAD51/G-135C* amplicons, digestions were performed with 10U of *Bst*NI (60ºC, 1 h). The genotypes G/G, G/C, or C/C were profiled as digestion products of 71/87, 71/87/157, and 157 bp bands, respectively. All samples were resolved on 3% agarose gels and visualized by ethidium bromide staining.

### Statistical analyses

Genotypic frequencies between patient/control groups were analyzed by Chi-Square Test and Odds Ratios with CI=95%. Contingency tables were used to compare groups concerning age, socio-economical and life style aspects by Chi-Square Test. Data were analyzed with Statistical Package 17.0 for Social Sciences (SPSS). Levels of significance *p*≤0.05, corresponding to 95.0% confidence, were considered in all analyses. All polymorphisms were regarded as potential markers by using the collective criteria of sensibility, specificity, positive and negative predictive values, and test accuracy.

## Results

### Population profiling and clinical records

Regarding age, there was no significant difference between patients (62.0±6.5) and control groups (61.6±10.3). Age-pairing between groups was essential concerning duration of exposure to environmental carcinogens. Case and control populations were also profiled similarly for basic items listed in Table S1 (geographic origin, education level, ethnic profile, familial cancer history, chronic diseases). When exposure to risk factors was weighted (tobacco, alcohol, or drug consumption), no significant differences were found between groups, with smokers and drinkers prevailing in both. Exposure to environmentally-borne agents (pesticides) was the only remarkable difference between case and control populations, and that caused a significant difference to appear (*p*<0.0001) towards the patient’s group. While 21.1% of patients declared having manipulated pesticides, only 3.5% of controls were subjected to this occupational hazard (Table 1).

**Table 1:** Exposure to occupationally-borne and other risk factors between case and control groups.

Risk Factors	Groups		p value
	Controls, n (%)	Cases, n (%)	
Organic solvents	43 (21.3)	9 (8.3)	
Chronic diseases	91 (45.7)	45 (41.3)	
Pesticides	7 (3.5)	23 (21.1) a	
Combustion products	5 (2.5)	10 (9.2)	*p < 0.0001*
Combustion + solvent	3 (1.5)	1 (0.9)	
None	50 (25.2)	21 (19.2)	
**Totals**	199* (100)	109* (100)	

Notes: p-value: descriptive level of the chi-square (Pearson). Only one p-value refers to a statistical model that indistinguishably compares all variables listed between control and patients’ groups.

a this particular environmental agent was highly significant (*p* < 0.05) in terms of risk for prostate cancer in exposed men.

* each patient was allocated in just one category, the one to which he declared to have been exposed to for longer periods.

The sampled populations were native to southeastern Brazil, mainly from the Rio de Janeiro metropolitan region (80%). The majority self-declared as ethnically white (53.8%), followed by brown or mulatto (30.7%) and black (15.5%). PSA values were obtained for 79 patients, ranging from 0.78-38.0 ng/mL (median 7.2 ± 5.4 ng/mL) (Table 2). Additional comparisons of SNP with age, prognosis, PSA level, and Gleason score unveiled two extra implications: (a) a Chi-Square significant association between *XPD* SNP and Gleason score (OR=9.31; 95%CI=1.19–428.0; *p*=0.022); (b) the *RAD51* SNP related with unclear prognosis (*p*=0.031).

**Table 2:** SNPs and clinic-pathological characteristics in prostate cancer patients. Results shown in bold were statistically significant.

	*XPD*			*RAD51*			*APEX1*		
Clinical record	wild-type	polymorphic	OR	wild-type	polymorphic	OR	wild-type	polymorphic	OR
			(95% CI)			(95% CI)			(95% CI)
Age (n=110)									
			0.82			0.47			0.90
			(0.32 - 2.04)			(0.17 - 1.28)			(0.37 - 2.21)
< 60	12(30%)	24(34.3%)	X^2^=0.212	24(28.6%)	12(46.2%)	X^2^=2.788	21(31.8%)	15(34.1%)	X^2^=0.062
			p=0.645			p=0.095			p=0.804
> 60	28(70%)	46(65.7%)		60(71.4%)	14(53.8%)		45(68.2%)	29(65.9%)	
Prognosis (n=43)			X^2^=3.055 p=0.217						
Good	1(11.2%)	5(14.3%)		5(13.9%)	1(14.3%)	**X^2^=6.954**	2(9.5%)	4(18.2%)	X^2^=0.897
Bad	4(44.4%)	24(68.6%)		26(72.2%)	2(28.6%)	*p=0.031*	15(71.4%)	13(59.1%)	p=0.638
Uncertain	4(44.4%)	6(17.1%)		5(13.9%)	4(57.1%)		4(19.1%)	5(22.7%)	
Gleason Score (n=57)									
			**9.31**			0.40			1.13
			**(1.19 – 428.0)**			(0.06 - 1.85)			(0.33 - 3.82)
			**X^2^=5.907**			X^2^=1.711			X^2^=0.053
< 7	15(93.8%)	25(61%)	*p=0.015*	25(56.8%)	10(76.9%)	p=0.191	20(64.5%)	16(61.5%)	p=0.816
≥ 7	1(6.2%)	16(39%)		19(43.2%)	3(23.1%)		11(35.5%)	10(38.5%)	
PSA (n=79)									
			1.74			0.53			0.57
			(0.54 - 5.46)			(0.17 - 1.64)			(0.19 - 1.69)
			X^2^=1.174			X^2^=1.600			X^2^=1.289
< 5ng/mL	9(39.1%)	15(26.8%)	p=0.278	14(25.9%)	10(40%)	p=0.206	12(25.5%)	12(37.5%)	p=0.256
≥ 5ng/mL	14(60.9%)	41(73.2%)		40(74.1%)	15(60%)		35(74.5%)	20(62.5%)	

### Prostate cancer risk and DNA repair genotyping

Genotypes of *XPD*, *RAD51,* and *APEX1* were determined for 110 patients and 200 controls. The most common allele was considered as the reference one, whereas the less common ones were grouped as variants (risk polymorphism) (Table 3). Neither *RAD51* (OR=1.13; 95%CI=0.64–1.96) nor *APEX1* polymorphic genes (OR=0.88; 95%CI=0.55–1.42) were associated with risk for PCa. Only for *XPD* (OR=2.36; 95%CI=1.46–3.84) a positive association was found for variant C/C or A/C genotypes. Wild-type RAD51 patients were significantly ascribed as the poorer prognosis (Table 2).

**Table 3:** Genotypic frequencies of *XPD*, *RAD51* and *APEX1* genotypes in 110 prostate cancer patients and 200 controls. Results shown in bold were statistically significant.

	*XPD*			*RAD51*			*APEX1*			Totals
	A|A	A|C	C|C	G|G	G|C	C|C	T|T	T|G	G|G	
Patients n (%)	40 (36.4)	55 (50.0)	15 (13.6)	84 (76.4)	24 (21.8)	2 (1.8)	66 (60.0)	33 (30.0)	11 (10.0)	110
										(100)
Control group n (%)	115 (57.5)	68 (34.0)	17 (8.5)	157 (78.5)	40 (20.0)	3 (1.5)	114 (57.0)	84 (42.0)	2 (1.0)	200 (100)
Totals	155 (50.0)	123 (39.7)	32 (10.3)	241 (77.7)	64 (20.6)	5 (1.6)	180 (58.1)	117 (37.7)	13 (4.2)	310 (100)
OR		**2.36**			1.13			0.88		
		**(1.46 – 3.84)**			(0.64 – 1.96)			(0.55 – 1.42)		
(95% CI)		***p*** **<0.001**			*p*=0.766			*p*=0.696		
Hardy-Weinberg Equilibrium		*p*=0.345			*p*=0.788			*p*=0.337		

n: number of individuals

Among the SNPs, the combined polymorphisms *XPD*/*RAD51* were found to have a harmful association (OR=3.40; 95% CI=1.32–9.20; *p*≤0.05). Other combinations such as *RAD51*/*APEX1* SNPs (OR=0.86; 95%CI=0.28–2.60), *XPD*/*APEX1* (OR=2.30; 95%CI=0.76–8.08), or *XPD*/*RAD51*/*APEX1* (OR=1.43; 95%CI=0.34–5.07) resulted in non-significant risks for PCa (Table S2).

In a comparative analysis with the International HapMap database (www.hapmap.org), only the *XPD* polymorphic allele and genotypic frequencies were statistically different in relation to Chinese (CHB) and Japanese (JPT) populations, calculated by the Chi-Square test (Table S3).The variant allele frequency ranged from 0.076 for Japanese (JPT) to 0.358 for Native American Indians (GIH).

In order to estimate whether these polymorphic genes could fulfill the role of diagnostic/prognostic markers, we performed a data performance diagnostic test for available genotypes. As shown in Table 4, the combination *XPD*/*RAD51* SNPs scored better for sensitivity, specificity, and accuracy.

**Table 4:** Data performance diagnostic test calculated for each polymorphism alone and combined

Parameters	Probabilities			
	*XPD*	*RAD51*	*APEX1*	*XPD + RAD51*
Sensitivity (%)	63.6 (54.3-72.0)	23.6 (16.7-32.4)	40.0 (31.3-49.3)	62.1 (44.0-77.3)
Specificity (%)	57.5 (50.5-64.1)	78.5 (72.3-83.6)	57.0 (50.0-63.7)	68.0 (54.2-79.2)
Positive predictive value (%)	45.2 (37.5-53.0)	37.7 (27.2-49.5)	33.8 (26.3-42.3)	52.9 (36.7-68.5)
Negative predictive value (%)	74.2 (66.7-80.4)	65.1 (58.9-70.8)	63.3 (56.1-70.0)	75.6 (61.3-85.7)
Accuracy (%)	59.7 (54.1-65.0)	59.0 (53.5-64.3)	50.1 (45.4-56.5)	65.8 (54.8-75.3)

## Discussion

The association between three dysfunctional SNPs (Asp148Glu/*APEX1*, G-135C/*RAD51* and Lys751Gln/*XPD*) and PCa susceptibility was evaluated in this case-control study. This is the first Brazilian study to cover SNPs in genes implicated in three main DNA repair pathways in a PCa population in Brazil. The present study was, thus, designed to improve the genetic profiling of PCa patients by mapping a panel of DNA repair SNPs and assess its correlation with clinical parameters. A comparative epidemiological inquiry on known cancer risk factors was applied to cases and controls. The collected data revealed that exposure to environmental factors, as well as the *XPD/*Lys751Gln (rs13181) polymorphism appeared to be associated with increased risk for PCa.

Major areas of research on PCa have focused on three main points: (1) assessment of how life style/environmental factors/diet can influence carcinogenesis; (2) strategies to delay disease onset and progression; and (3) finding accurate biomarkers to distinguish between indolent and aggressive forms (Shen and Abate-Shen, 2010). The present study was designed to target items (1) and (3).

### The roles of *XPD* and *RAD51* SNPs in prostate cancer proneness

A significantly increased risk for lymphohematopoietic, prostate, melanoma, and brain neoplasms has been surveyed among pesticide handlers and industry workers (Koutros *et al.*, 2010). Moreover, the NER pathway was recently shown to repair damage induced by carcinogens, including pesticides (Barry *et al.*, 2012). Accordingly, data from the present study associated an NER dysfunction (*XPD*) with high risk for PCa (Table 3), especially in men exposed to pesticides. When risk factors were compared between cases and controls, a remarkable positive correlation was found only for exposure to pesticides. The expressive amount of *XPD* AC and CC polymorphic patients exposed to pesticides implies that they can steeply accumulate damages because of NER malfunction, leading to increased PCa risk.

Extensive DNA damage was detected in exposed floriculturists compared to non-exposed ones, and exposure to pesticides appeared as the primary genotoxic factor for long-term carcinogenesis (Bolognesi *et al.*, 2004). Theophilou *et al.* (2015) followed-up transgenerational alterations in human prostate tissue samples over 30 years, correlated with geographic distribution and exposure to risk factors. Indeed, genotoxic agents seem to ensue long-lasting genetic/epigenetic alterations, imprinting phenotypic pro-malignant changes.

Although the association of lifestyle and cancer risk had already been highlighted in a number of studies, none of the other noxious habits (smoking, drug use), or social aspects could be ascribed to PCa risk in the studied group. Here, only the *XPD* SNP was related to the risk of developing PCa. It is worthy of note that *XPD*-mediated NER corrects damages caused by environmental agents and reactive oxygen species (Mitra *et al.*, 2001), these being important triggers of PCa. This supports the complex genetic basis of PCa involving multiple susceptibility genes.

*Sulfolobus acidocaldarius* XPD (SaXPD protein) harbors a catalytic site for its SaRAD51 protein, and all SaXPD helicase domains were conserved in human XPD (Fan and Wilson, 2005). Four domains comprise the SaXPD’s catalytic core: two RAD51/RecA-like domains (HD1-HD2) and two additional HD1 domains inserted together. Twenty-two out of the known 26 point mutations related to XPD-linked human diseases mapped there (Xeroderma pigmentosum, Cockayne syndrome, and Trichothiodystrophy). Aloyz *et al.* (2002) showed that the physical association between human XPD and RAD51 proteins acting to remove DNA crosslinks maybe assembled in a larger complex. They appear to be recombination counterparts, modulating the HRR-mediated resistance to crosslinks.

By playing an important role in recovering damaged DNA in vertebrates, the RAD51 hyper-recombination phenotype can potentiate genomic instability (Klein, 2008). Schild and Wiese (2010) suggest that *RAD51* overexpression can contribute to carcinogenic progression. Since XPD and RAD51 are counterparts in the repair of DNA breaks, their double malfunctions appeared statistically related to high risk for PCa, perhaps because of the triggered genomic instability. Noteworthy, wild-type RAD51 patients underwent worse prognoses, probably because of better repair of therapy-induced DNA damages by HRR, while XPD/Lys751Gln patients displayed higher Gleason scores (Table 2). Despite the small number of cases analyzed, these correlations were highly significant.

Regarding general clinical findings, both specificity and sensitivity tests also revealed that *XPD* and *XPD*+*RAD51* genotypes scored good negative predictive values (Table 4). Regarding the potential validity of our proposed panel, since *XPD* and *RAD51* SNPs significantly scored the top OR in a small population, they would probably reach a much more relevant status as risk markers when assessing a larger group.

A Spanish multicenter group revealed that genes involved in HRR could be associated with poor prognosis in PCa (Henriquez-Hernandez *et al.*, 2014). Their findings corroborate ours, regarding the association of HRR SNPs with unclear prognosis. The same group reported that DNA repair genetic variants (*XRCC6* and *MVP*) were associated with more aggressive outcomes (Henriquez-Hernandez *et al.*, 2016).

Our genetic analysis is endorsed by the HapMap database, *i.e.*, the Southeast Brazilian population is quite different from the Japanese and Chinese. The European colonization remains strongly imprinted in Brazilian ethnicity, remaining genetically distant from Eastern populations. Accordingly, the genotypic profiling seen by Henriquez-Hernandez *et al.* (2014) in a Spanish population and a meta-analysis performed by Mi *et al.* (2012) with Asian and African populations matches our HapMap assessments. Studies about ethnically-linked differences strengthen the need to continue research on new confident biomarkers.

### The *APEX1* SNP counteracting risky genotypes

Interestingly, the *APEX1* deficiency caused the risk for PCa to drop (Table S2), and this result is interpreted as deriving from the simultaneous knockdown of three key routes of DNA repair: HRR, NER, and BER. This probably drives prostatic cells to death, protecting the prostatic tissue from the outbreak of potentially malignant clones.

The *APEX1/*Asp148Glu (rs1130409) SNP was recorded in another Brazilian population (Kuasne *et al.*, 2011), which appeared to be more susceptible to PCa. In our group, however, no susceptibility was connected with this SNP, maybe because of other genetic background differences between the sampled populations and the low penetrance of this SNP for cancer development.

### Genetic instability assessment and decision making in prostate cancer

Data compiled for this Brazilian group revealed the need for a clinical follow-up, along with a panel of key DNA repair dysfunctions, instead of just one or two genes. Moreover, biomarker research has mainly targeted diagnosis, rather than prognosis and outcomes (Prensner *et al.*, 2012), which are capable of tailoring individual therapeutics. For instance, Chantre-Justino *et al.* (2015) reported that patients with lower transcript levels of the *APE1* gene belonged to the cohort with 100% lethality from aggressive bladder cancer. In prostate cancer, the expression of a panel of 17 genes belonging to nine different DNA damage and repair (DDR) pathways was determined in prostatectomy samples in a cohort study by Evans *et al.* (2016), where high expression of NER and BER genes significantly related to metastasis and lower overall survival. The authors envisaged the screening of individual “DDR signatures” to track aggressive prostate cancers on the basis of their differential response to therapy. These two studies on genetic markers in tumors of two urinary-related organs point out how tissue-specific the neoplastic process can be driven.

In this study, we observed that patients with a polymorphic *XPD* genotype significantly ranked at higher Gleason scores, indicating that NER-deficient individuals seem to bear genetically more instable tumors. In the absence of the error-free NER pathway, other concurrent error-prone mechanisms could take control of less accurately repair of DNA damages. In support of this finding, *XPD* SNPs rs13181 and rs1799793 (*XPD*/Asp312Asn) tended to group under high Gleason scores and advanced tumor stages, although results were not statistically significant (Agalliu *et al.*, 2010). Altogether, these findings help to unveil genetic features on how this disease is triggered and progresses in different populations, whose heterogeneity can render even more complex outcomes due to gene-environment interactions. Present findings await corroboration by other studies, focusing on environmental and ethnic factors impacting this important male cancer.

Complimentary tests, such as ultrasonography and biopsy should be included in more precise diagnostics, although the lower cutoff of 4.1 ng/ml of PSA protein, for example, is consensually taken as a good negative prediction (Van den Broeck *et al.*, 2015). If early cancer detection by current tests is the desired target in cancer prevention, genotyping instability-prone DNA repair SNPs can be envisaged as the earliest informative test amongst all.

## Supplementary material

The following online material is available for this article:

Table S1 - Distribution of patients and controls among different socio-economical and life style groups.

Table S2 - Frequencies of the combined genotypes in 110 prostate cancer patients and 200 controls.

Table S3 - Allelic and genotype frequencies of *XPD* Lys751Gln in HapMap and Brazilian populations.

## References

[B1] Agalliu I, Kwon E, Salinas C, Koopmeiners JS, Ostrander EA, Stanford JL (2010). Genetic variation in DNA repair genes and prostate cancer risk:
Results from a population-based study. Cancer Causes Control.

[B2] Aloyz R, Xu ZY, Bello V, Bergeron J, Han FY, Yan Y, Malapetsa A, Alaoui-Jamali M, Duncan A, Panasci L (2002). Regulation of cisplatin resistance and homologous recombinational
repair by the TFIIH subunit XPD. Cancer Res.

[B3] Antoniou AC, Sinilnikova OM, Simard J, Léoné M, Dumont M, Neuhausen SL, Struewing JP, Stoppa-Lyonnet D, Barjhoux L, Hughes DJ (2007). RAD51 135G > C modifies breast cancer risk among BRCA2
mutation carriers: Results from a combined analysis of 19
studies. Am J Hum Genet.

[B4] Baccarelli A, Calista D, Minghetti P, Marinelli B, Albetti B, Tseng T, Hedayati M, Grossman L, Landi G, Struewing JP (2004). *XPD* gene polymorphism and host characteristics in the
association with cutaneous malignant melanoma risk. Br J Cancer.

[B5] Barry KH, Koutros S, Andreotti G, Sandler DP, Burdette LA, Yeager M, Beane Freeman LE, Lubin JH, Ma X, Zheng T (2012). Genetic variation in nucleotide excision repair pathway genes,
pesticide exposure and prostate cancer risk. J Carcinog.

[B6] Bolognesi C, Landini E, Perrone E, Roggieri P (2004). Cytogenetic biomonitoring of a floriculturist population in
Italy: Micronucleus analysis by fluorescence in situ (FISH) with
all-chromosome centromeric probe. Mutat Res.

[B7] Bostwick DG, Burke HB, Djakiew D, Euling S, Ho SM, Landolph J, Morrison H, Sonawane B, Shifflett T, Waters DJ (2004). Human Prostate Cancer Risk Factors. J Am Cancer Soc.

[B8] Chantre-Justino M, Alves G, Britto C, Cardoso A, Scherrer L, Moreira Ados S, Quirino R, Ornellas A, Leitão A, Lage C (2015). Impact of reduced levels of APE1 transcripts on the survival of
patients with urothelial carcinoma of the bladder). Oncol Rep.

[B9] Chen Y, Li J, Mo Z (2016). Association between the APEX1 Asp148Glu polymorphism and prostate
cancer, especially among Asians: A new evidence-based
analysis. Oncotarget.

[B10] Evans JR, Zhao SG, Chang SL, Tomlins SA, Erho N, Sboner A, Schiewer MJ, Spratt DE, Kothari V, Klein EA (2016). Patient-level DNA damage and repair pathway profiles and
prognosis after prostatectomy for high-risk prostate cancer. JAMA Oncol.

[B11] Fan J, Wilson DM (2005). Protein-protein interactions and post translational modifications
in mammalian base excision repair. Free Radic Biol Med.

[B12] Galkin VE, Wu Y, Zhang XP, Qian X, He Y, Yu X, Heyer WD, Luo Y, Egelman EH (2006). The Rad51/RadA N-terminal domain activates nucleoprotein filament
ATPase activity. Structure.

[B13] Gangwar R, Manchanda PK, Mittal RD (2009). Implications of XRCC1, XPD and APE1 gene polymorphism in North
Indian population: A comparative approach in different ethnic groups
worldwide. Genetica.

[B14] Henríquez-Hernández LA, Valenciano A, Foro-Arnalot P, Álvarez-Cubero MJ, Cozar JM, Suárez-Novo JF, Castells-Esteve M, Fernández-Gonzalo P, De-Paula-Carranza B, Ferrer M (2014). Single nucleotide polymorphisms in DNA repair genes as risk
factors associated to prostate cancer progression. BMC Med Genet.

[B15] Henríquez-Hernández LA, Valenciano A, Foro-Arnalot P, Álvarez-Cubero MJ, Cozar JM, Suárez-Novo JF, Castells-Esteve M, Fernández-Gonzalo P, De-Paula-Carranza B, Ferrer M (2016). Association between single-nucleotide polymorphisms in DNA
double-strand break repair genes and prostate cancer aggressiveness in the
Spanish population. Prostate Cancer Prostat Dis.

[B16] Hu JJ, Smith TR, Miller MS, Mohrenweiser HW, Golden A, Case LD (2001). Amino acid substitution variants of APE1 and XRCC1 genes
associated with ionizing radiation sensitivity. Carcinogenesis.

[B17] INCA, Brazilian National Cancer Institute (2016). Estimate 2015-2016. Cancer Incidence in Brazil.

[B18] Klein HL (2008). The consequences of Rad51 overexpression for normal and tumor
cells. DNA Repair.

[B19] Koutros S, Alavanja M, Freeman L (2010). An update of cancer incidence in the agricultural health
study. J Occup Environ Med.

[B20] Kuasne H, Rodrigues IS, Losi-Guembarovski R, Reis MB, Fuganti PE, Gregório EP, Libos F, Matsuda HM, Rodrigues MA, Kishima MO (2011). Base excision repair genes XRCC1 and APEX1 and the risk for
prostate cancer. Mol Biol Rep.

[B21] Levy-Lahad E, Lahad A, Eisenberg S, Dagan E, Paperna T, Kasinetz L, Catane R, Kaufman B, Beller U, Renbaum P (2001). A single nucleotide polymorphism in the RAD51 gene modifies
cancer risk in BRCA2 but not BRCA1 carriers. Proc Natl Acad Sci USA.

[B22] Majumder M, Sikdar N, Ghosh S, Roy B (2007). Polymorphisms at XPD and XRCC1 DNA repair loci and increased risk
of oral leukoplakia and cancer among NAT2 slow acetylators. Int J Cancer.

[B23] Mi Y, Zhang L, Feng N, Wu S, You X, Shao H, Dai F, Peng T, Qin F, Zou J (2012). Impact of two common xeroderma pigmentosum group D (XPD) gene
polymorphisms on risk of prostate cancer. PLoS One.

[B24] Mitra S, Boldogh I, Izumi T, Hazra TK (2001). Complexities of the DNA base excision repair pathway for repair
of oxidative DNA damage. Environ Mol Mutagen.

[B25] O’Donovan A, Wood RD (1993). Identical defects in DNA repair in xeroderma pigmentosum group G
and rodent ERCC group 5. Nature.

[B26] Platz EA, Giovannucci E, Schottenfeld D, Fraumeni JF (2006). Prostate cancer. Cancer Epidemiology Biomarkers & Prevention.

[B27] Pramanik S, Devi S, Chowdhary S, Surendran ST, Krishnamurthi K, Chakrabarti T (2011). DNA repair gene polymorphisms at XRCC1, XRCC3, XPD and OGG1 loci
in Maharashtrian population of central India. Chemosphere.

[B28] Prensner J, Rubin M, Wei J, Cinnalyan AM (2012). Beyond PSA: The next generation of prostate cancer
biomarkers. Sci Transl Med.

[B29] Ramana CV, Boldogh I, Izumi T, Mitra S (1998). Activation of apurinic/apyrimidinic endonuclease in human cells
by reactive oxygen species and its correlation with their adaptive response
to genotoxicity of free radicals. Proc Natl Acad Sci USA.

[B30] Sambrook J (1989). Molecular Cloning: A Laboratory Manual.

[B31] Schild D, Wiese C (2010). Overexpression of RAD51 suppresses recombination defects: A
possible mechanism to reverse genomic instability. Nucleic Acids Res.

[B32] Shen MM, Abate-Shen C (2010). Molecular genetics of prostate cancer: New prospects for old
challenges. Genes Dev.

[B33] Theophilou G, Lima KM, Briggs M, Martin-Hirsch PL, Stringfellow HF, Martin FL (2015). A biospectroscopic analysis of human prostate tissue obtained
from different time periods points to a trans-generational alteration in
spectral phenotype. Sci Rep.

[B34] Van den Broeck T, De Ridder D, Van der Aa F (2015). The value of surgical release after obstructive anti-incontinence
surgery: An aid for clinical decision making. Neurourol Urodyn.

[B35] Vettriselvi V, Vijayalakshmi K, Solomon PF, Venkatachalam P (2007). XRCC1 and XPD gene polymorphisms in a South Indian
population. Asian Pac J Cancer Prev.

[B36] Wang WW, Spurdle AB, Kolachana P, Bove B, Modan B, Ebbers SM, Suthers G, Tucker MA, Kaufman DJ, Doody MM (2001). A single nucleotide polymorphism in the 5’ untranslated region of
RAD51 risk of cancer among BRCA1/2 mutation carriers. Cancer Epidemiol Biomarkers Prev.

[B37] White M (2009). Structure, function and evolution of the XPD family of
iron-sulfur containing 5’ → 3’ DNA helicases. Biochem Soc Trans.

