# Supplementary material for: Relationship between XPD, RAD51, and APEX1 DNA repair genotypes and prostate cancer risk in the male population of Rio de Janeiro, Brazil
Source: Genet Mol Biol. 2017 Nov 6;40(4):751–8. doi: 10.1590/1678-4685-GMB-2017-0039 (PMC5738611; doi:10.1590/1678-4685-GMB-2017-0039)
Supplement: Supplementary file 1 [file 1415-4757-gmb-1678-4685-GMB-2017-0039-Suppl01.pdf]

Supplementary Material to "Relationship between *XPD, RAD51,* and *APEX1* DNA repair genotypes and prostate cancer risk in the male population of Rio de Janeiro, Brazil"

Table S1 - Distribution of patients and controls among different socio-economical and life style groups.

Parameter		Groups				p value
		Controls		Cases		
		N	%	N	%	
**Smoking**						
	No	76	38.0	32	29.1	0.135
	Yes	124	62.0	78	70.9	
**Alcoholism**						
	No	38	19.0	18	16.4	0.644
	Yes	162	81.0	92	83.6	
**Geografic origin in Brazil**	North	2	1.0	6	5.5	
	Northeast	27	13.5	17	15.5	
	Southeast	166	83.0	85	77.3	0.094
	Midwest	0	0.0	1	0.9	
	South	2	1.0	1	0.9	
	Other	3	1.5	0	0.0	
**Education level**						
	Illiterate	1	0.5	0	0.0	
	Incomplete school	65	32.5	45	40.9	
	Complete school	59	29.5	19	17.3	
	Incomplete high school	12	6.0	4	3.6	
	Complete high school	33	16.5	19	17.3	0.204
	Technical	10	5.0	10	9.1	
	Incomplete College	19	9.5	13	11.8	
	Complete College	1	0.5	0	0.0	
**Ethnicity**						
	White	101	50.5	66	60.0	
	Mulato	65	32.5	30	27.3	0.264
	Black	34	23.5	14	12.7	
**Familial cancers**						
	No	108	54.0	60	54.5	
	Yes	92	46.0	50	45.5	1.000
**Drug addiction**						
	No	192	96.0	105	95.5	
	Yes	8	4.0	5	4.5	0.776
Totals:		200	100.0	110	100.0	
