# Supplementary material for: Relationship between XPD, RAD51, and APEX1 DNA repair genotypes and prostate cancer risk in the male population of Rio de Janeiro, Brazil
Source: Genet Mol Biol. 2017 Nov 6;40(4):751–8. doi: 10.1590/1678-4685-GMB-2017-0039 (PMC5738611; doi:10.1590/1678-4685-GMB-2017-0039)
Supplement: Supplementary file 2 [file 1415-4757-gmb-1678-4685-GMB-2017-0039-Suppl02.pdf]

Genetics and Molecular Biology

Supplementary Material to "Relationship between *XPD, RAD51,* and *APEX1* DNA repair genotypes and prostate cancer risk in the male population of Rio de Janeiro, Brazil"

Table S2 - Frequencies of the combined genotypes in 110 prostate cancer patients and 200 controls.

Genotypes		Patients		Controls		OR (95% CI)
		N	%	N	%	
XPD and *RAD51*	**Reference genotype:**					**3.40**
	XPD: A/A and *RAD51*: G/G	11	37.93	34	68.00	
	**Other genotypes:**					**(1.32 - 9.20)**
	XPD: C/C or A/C and *RAD51*: C/C or G/C	18	62.07	16	32.00	**p<0.05**
RAD51 and *APEX1*	**Reference genotype:**					0.86
	RAD51: G/G and *APE1*: T/T	30	78.95	29	76.32	
	**Other genotypes:**					(0.28 - 2.60)
	RAD51: C/C or G/C and *APE1*: G/G or T/G	8	21.05	9	23.68	p=1.000
XPD and *APEX1*	**Reference genotype:**					2.30
	XPD: A/A and *APE1*: T/T	5	15.63	14	30.43	
	**Other genotypes:**					(0.76 – 8.08)
	XPD: C/C or A/C and *RAD51*: C/C or G/C	27	84.38	32	69.57	p=0.216
XPD, RAD51 and *APEX1*	**Reference genotype:**					1.43
	XPD: A/A and *RAD51*: G/G and *APE1*: T/T	18	81.82	57	86.36	(0.34 – 5.07)
	**Other genotypes:**					
	XPD: C/C or A/C and *RAD51*: C/C or G/C and *APE1*: G/G or T/G	4	18.18	9	13.64	p=0.831
