# Supplementary material for: Relationship between XPD, RAD51, and APEX1 DNA repair genotypes and prostate cancer risk in the male population of Rio de Janeiro, Brazil
Source: Genet Mol Biol. 2017 Nov 6;40(4):751–8. doi: 10.1590/1678-4685-GMB-2017-0039 (PMC5738611; doi:10.1590/1678-4685-GMB-2017-0039)
Supplement: Supplementary file 3 [file 1415-4757-gmb-1678-4685-GMB-2017-0039-Suppl03.pdf]

Supplementary Material to "Relationship between *XPD, RAD51,* and *APEX1* DNA repair genotypes and prostate cancer risk in the male population of Rio de Janeiro, Brazil"

Table S3 - Allelic and genotype frequencies of *XPD* Lys751Gln in HapMap and Brazilian populations.

POPULATION	Genotypic and Allelic Frequencies of *XPD*										p value of χ2 of Pearson in genotype comparison between Brazilian population and different HapMap populations
	GENOTYPES						ALLELIC FREQUENCY				
	Genotype	Frequency (No)	Genotype	Frequency (No)	Genotype	Frequency (No)	Wild Type	Frequency (No)	Wild Type	Frequency (No)	
CEU (n=113)	T/T	0.407 (46)	G/T	0.522 (59)	G/G	0.071 (8)	T	0.668	G	0.332	0.065
CHB (n=84)	T/T	0.798 (67)	G/T	0.202 (17)	G/G	0.0 (0)	T	0.899	G	0.101	**< 0.001**
GIH (n=88)	T/T	0.398 (35)	G/T	0.489 (43)	G/G	0.114 (10)	T	0.642	G	0.358	0.228
JPT (n=86)	T/T	0.860 (74)	G/T	0.128 (11)	G/G	0.012 (1)	T	0.924	G	0.076	**< 0.001**
Sind (n=255)	T/T	0.51 (130)	G/T	0.408 (96)	G/G	0.082 (21)	T	0.71	G	0.29	0.698
Nind (n=209)	T/T	0.435 (91)	G/T	0.46 (96)	G/G	0.105 (40)	T	0.665	G	0.335	0.322
Elnd (n=388)	T/T	0.49 (190)	G/T	0.407 (158)	G/G	0.103 (40)	T	0.69	G	0.31	0.959
MAH (n=215)	T/T	0.51 (110)	G/T	0.377 (81)	G/G	0.111 (24)	T	0.7	G	0.3	0.883
Brazil (n=310)	T/T	0.50 (155)	G/T	0.396 (123)	G/G	0.103 (32)	T	0.7	G	0.3	-------

[a]Chi-square test of Pearson more than 5% significance level, so populations are not significantly different from Brazil.
[b]Population data source: HapMap (www.hapmap.org).

Population nomenclature:
CEU-Utah residents with Northern and Western European ancestry from the CEPH collection.
CHB-Han Chinese in Beijing, China.
GIH-Gujarati Indians in Houston, Texas.
JPT-Japanese in Tokyo, Japan.
SInd-South Indian population in India (Vettriselvi et al., 2007).
NInd-North Indian population in India (Gangwar et al., 2009).
EInd-Eastern Indian population from Calcutta, India (Majumder et al., 2007).
MAH-Maharastrian population residing in Vidarbha region of central India.
